# An Array SPRi Biosensor for the Determination of Follicle-Stimulating Hormone in Blood Plasma

**DOI:** 10.3390/s23249686

**Published:** 2023-12-07

**Authors:** Anna Sankiewicz, Beata Zelazowska-Rutkowska, Zenon Lukaszewski, Adam Hermanowicz, Ewa Gorodkiewicz

**Affiliations:** 1 Bioanalysis Laboratory, Faculty of Chemistry, University of Bialystok, 15-245 Bialystok, Poland; ewka@uwb.edu.pl; 2 Department of Pediatric Laboratory Diagnostics, Medical University of Bialystok, 15-274 Bialystok, Poland; zelazowskab@wp.pl; 3 Faculty of Chemical Technology, Poznan University of Technology, 60-965 Poznan, Poland; zenon.lukaszewski@put.poznan.pl; 4 Department of Pediatric Surgery and Urology, Medical University of Bialystok, 15-274 Bialystok, Poland; ahermanowicz@wp.pl

**Keywords:** liquid biopsy, FSH, array SPRi, blood plasma, immunosensor

## Abstract

Follicle-stimulating hormone (FSH) regulates the development, growth, pubertal maturation and reproductive processes of the human body. The determination of serous FSH concentration is significant as an alternative to testicular biopsy in the case of boys suffering from cryptorchidism after orchidopexy, and as a means of determining the menopausal stage in women. The aim of this investigation is to develop a specific array surface plasmon resonance imaging (SPRi) biosensor for the determination of FSH in body liquids such as blood plasma, obtaining sufficient sensitivity to determine FSH at levels characteristic for that hormone in blood plasma, without any signal enhancement. The biosensor consists of a mouse monoclonal anti-FSH antibody attached to the gold surface of a chip via a cysteamine linker. Its linear response range is from 0.08 mIU mL^−1^ (LOQ) to 20 mIU mL^−1^, and well covers most of the range of FSH activities found in blood without dilution. The precision of measurement is between 3.2% and 13.1% for model samples, and between 3.7% and 5.6% for spiked plasma samples. Recoveries are in the range from 94% to 108%. The biosensor has good selectivity, and is validated by comparison with ECLE, with good agreement of the results

## 1. Introduction

Follicle-stimulating hormone (FSH) regulates the development, growth, pubertal maturation and reproductive processes of the human body. It stimulates Graafian follicle growth in females, and its cyclical increase and decrease are essential for monthly ovulation [1]. FSH promotes the development of the testes and the differentiation of sperm in males [2]. During menopause, the FSH level increases and remains stable [3]. Higher FSH levels cause more frequent hot flushes and night sweats and increase the level of circulating low-density lipoprotein (LDL) cholesterol. The determination of FSH is an alternative to testicular biopsy in boys suffering from cryptorchidism after orchidopexy. Such biopsies are not always desirable or accepted, and the serous FSH level can be used to evaluate the risk of infertility and the need for adjuvant hormonal treatment [4,5]. The extragonadal action of FSH has been described in studies. FSH can play an important role in metabolic diseases [6,7], lipogenesis, insulin sensitivity [8], inflammation [9] and cancer [10,11].

FSH belongs to the group of gonadotropic hormones and is produced by the pituitary gland and released into the blood [3]. It is a glycoprotein heterodimer of 35.5 kDa with two polypeptide units: α and β, where α subunits consist of 96 aminoacids and β subunits of 111 aminoacids. Each subunit has two N-linked glycosylation sites, which are on Asn 52 and Asn 78 cites for the α-subunit and Asn 7 and Asn 24 for the β-subunit [12]. In a given species, gonadotropic hormones share an identical amino acid sequence of the α-chain. Their functional specificity is derived from different β-chains. FSH acts by binding to G protein-coupled receptors that signal, in part, through the protein kinase A pathway [13].

Serum FSH concentration is usually expressed in mIU mL-1 or IU L^−1^ and strongly depends on sex and age. Normal adult male serum concentration ranges between 3.0 and 9.5 mIU mL^−1^ FSH [14]. Lower concentrations are found in boys (0.5–3.7 mIU mL^−1^) and males aged over 60 (9–14 mIU mL^−1^) [15]. Serous FSH concentrations in girls and women are usually slightly higher than or equal to those found in boys or men of the same age (1.3–4.4 mIU mL^−1^), with the exception of menopausal and post-menopausal ages, when a large rise in serous FSH concentration is observed [15].

Abnormal FSH levels may be related with primary gonadal dysfunction, post menopause, polycystic ovary syndrome, pituitary adenoma in females and with hypogonadism, and primary testicular failure in men. Measuring the serum FSH is particularly useful for differentiation of primary or secondary hypogonadism [16]. Primary hypogonadism is characterized by low serum testosterone and high luteinizing hormone (LH) and FSH concentrations. The cause of primary hypogonadism can be testicular injury, tumor, or infection or genetic defects affecting testicular development (e.g., Klinefelter syndrome), as well as chemotherapy, radiation treatment. In contrast, secondary hypogonadism is characterized by low LH and FSH leading secondarily to low testosterone. Secondary hypogonadism can be caused by hypothalamic and pituitary disorders or lesions, hyperprolactinemia or genetic defects causing disorders of gonadotropin synthesis (Kallmann syndrome) [17].

Ljubicic et al. [15] reported FSH concentrations of 50–65 immunoradiometric assay for that period, while Kawakita et al. [18] reported 70–124 mIU mL^−1^. According to Itoh et al. [14], the reported FSH concentration depends on the analytical method used for measurement. Several methods have been used for serous FSH determination: immunoradiometric assay [14], double antibody radioimmunoassay [14], fluoroimmunoassay [1,15], electrochemiluminescence immunoassay [4] and chemiluminescent immunoassay [18], capillary electrophoresis with enhanced chemiluminescence detection [19], stochastic biosensor [20]. The analytical parameters of example methods for determining FSH are presented in Table 1. The lack of a gold standard in the determination of FSH is a good argument for developing new methods for this purpose.

Array SPRi has the potential to compete with the above-mentioned methods. In contrast to most of them, array SPRi is a label-free technique, and uses biosensors with a very simple structure. Array SPRi enables the determination of a great variety of biomarkers without signal enhancement or analyte preconcentration [22]. Array SPRi differs significantly from the commonly used fluidic version of SPRi or SPR. In fluidic SPRi, the biosensor is formed in situ during the measurement, while in array SPRi, the biosensor is prepared ex situ before measurement. Another significant difference is that in the fluidic version, the measurement is performed in the presence of the processing liquid, but in array SPRi, it takes place with the removal of the processing liquid. An additional advantage of array SPRi is the possibility of simultaneous determination of numerous samples due to the array chip architecture. More than 30 biomarkers can be determined in blood serum or plasma with array SPRi, including hormones [23] and cancer biomarkers such as CEA, CA 125, HE 4. For example, CEA can be determined by array SPRi at levels characteristic for healthy persons or cancer patients, using a very simple biosensor consisting of just an antibody and an appropriate linker [24]. Fluidic SPR requires the use of a sandwich structure and gold nanoparticles for signal enhancement [25] to achieve a similar effect. The SPR method in the classic version was used to detect FSH [21]. Array SPRi with suitable biosensors has been successfully used in numerous clinical investigations [26,27,28].

The aim of this investigation was to develop a specific array SPRi biosensor for the determination of FSH in body liquids such as blood plasma, obtaining sufficient sensitivity to determine FSH at levels characteristic for that hormone in blood plasma, without any signal enhancement. In a preliminary investigation, it was found that a mouse monoclonal anti-FSH antibody attached to the gold surface of a chip via cysteamine linker may enable the achievement of this goal.

## 2. Materials and Methods

### 2.1. Materials and Reagents

Recombinant human FSH α/β (R&D Systems, Minneapolis, MN, USA), mouse monoclonal anti-FSH antibody, recombinant human thyroid stimulating hormone (TSH) protein, (ABCAM, Cambridge, UK), testosterone, luteinizing hormone from human pituitary (LH), cysteamine hydrochloride, N-ethyl- N’-(3-dimethylaminopropyl) carbodiimide (EDC), N-hydroxysuccinimide (NHS) (SIGMA-ALDRICH, Munich, Germany), absolute ethanol, acetic acid, hydrochloric acid, sodium hydroxide, sodium chloride, sodium carbonate, sodium acetate (POCh, Gliwice, Poland), HBSES buffer pH = 7.4 (0.01 M HEPES, 0.15 M sodium chloride, 0.005% Tween 20, 3 mM EDTA), Phosphate Buffered Saline (PBS) pH = 7.4, and carbonate buffer pH = 8.5 (BIOMED, Lublin, Poland) were used. Argon N 5.0 with content Ar ≥ 99,999% was used (AIR LIQUIDE Polska Sp. z o.o., Krakow, Poland). The base of the biosensor was a chip covered with a layer of gold (Ssens, Enschede, The Netherlands).

### 2.2. Biological Materials

The biological material used in the research consisted of blood plasma samples from healthy boys with cryptorchidism, without any chronic disease, and healthy boys with both testes in the scrotum, admitted for elective surgery of inguinal hernia. The samples were obtained from the Department of Pediatric and Urologic Surgery at the Medical University of Bialystok (Poland). The blood was collected in EDTA tubes on the morning of the surgery and was then centrifuged. The plasma was frozen at −80 °C.

The study was approved by the local ethics committee of the Medical University of Bialystok (Poland) (R-I-002//152/2014, R-I-002/178/2009). All parents provided written, informed consent for this study.

The tested samples were not diluted.

### 2.3. SPRi Apparatus

The SPRi spectrometer used in the study was constructed in the Bioanalysis Laboratory of the University of Bialystok. The details of the construction are given in a previous article [29]. The device consists of, among other components, a He-Ne laser, two glass lenses L1 (f 3 mm) and L2 (f 300 mm), two polarizers (P1 and P2), a mirror, a glass prism, and a CCD camera. The Kretschman configuration was used for the measurements. The biosensor was prepared before the measurement and was placed on a prism. The SPRi signal was measured twice, after the immobilization of the antibody and then after interaction with a solution containing FSH. The measurement was performed at a constant light angle. Images were recorded using a CCD camera and the SPRi signal was calculated using ImageJ software (NIH, version 1.32).

### 2.4. Reference Technique

The electrochemiluminescence technique (Elecsys FSH) performed on a COBAS E-411 instrument was used for the validation of the developed method. The quantitation was performed according to the recommendations of the manufacturer’s protocol.

### 2.5. Biosensor Preparation

The biosensors were based on glass chips with sputtered gold, purchased directly from the manufacturer (SSens, Enschede, The Netherlands). Before coating with successive biosensor layers, the gold chip must be chemically modified. The first step was thiol anchoring. The gold plate was immersed in a 20 mM alcoholic cysteamine solution for a minimum of 12 h at room temperature. After the deposition of thiol on the surface of the gold, the chip was rinsed in anhydrous ethyl alcohol and water. The next step was the attachment to the modified chip of a molecule (FSH antibody) that has an affinity with the target analyte (FSH protein). This was achieved by means of covalent immobilization of the antibody via EDC/NHS esters. The antibody solution was activated with a 1:1 mixture of NHS (250 mM) and EDC (250 mM) in a buffer solution (pH 8.5). Next, 3 µL of an activated antibody was placed on the amine-modified surface. After incubation for 1 h at 37 °C, the chip was washed with water. To eliminate nonspecific adsorption, after the incubation of the chip with the antibody, a bovine serum albumin (BSA) solution with a concentration of 1 ng mL^−1^ was applied to the biosensor’s active sites, and it was then washed several times with water and dried under a stream of argon. Following these procedures, the biosensor was capable of capturing FSH from a solution. Now, 3 µL of the investigated solution was applied to the active sites of the biosensor. The ligand–analyte interaction time was 10 min. After this time, the surface of the active sites of the biosensor was rinsed with an HBS-ES solution and distilled water and removed. All the biochemical reactions were carried out in the presence of phosphate buffer saline (PBS) at pH = 7.40. During preparation of the biosensor, the surface was controlled by scanning electron microscope (SEM). See also Supplementary Materials Figure S1.

## 3. Results

### 3.1. Optimization of Antibody Concentration

**Figure 1:**
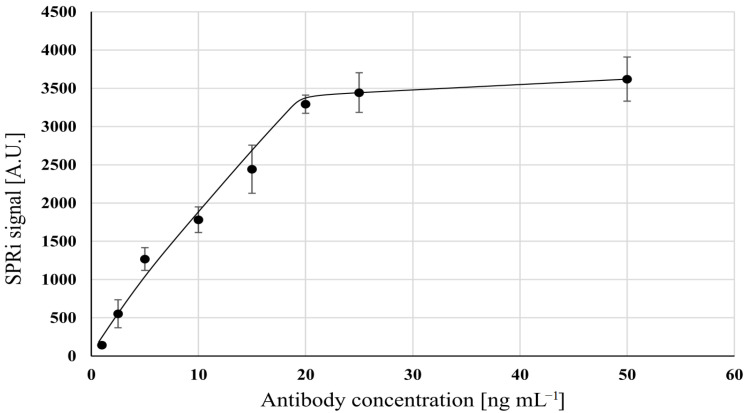
Optimization of mouse monoclonal anti-FSH antibody concentration. FSH activity: 31.5 mIU mL^−1^; pH = 7.4. Error bars were calculated for 12 independent measurements for each concentration at a 95% confidence level.

The concentration of antibody solution determines the number of active points of the biosensor and should correspond to the expected FSH concentration range. Optimization experiments were performed in a PBS buffer (pH 7.4) at a constant FSH activity (31.5 mIU mL^−1^). In a series of experiments, biosensors were formed using different concentrations of a mouse monoclonal anti-FSH antibody. These biosensors were tested at a constant FSH activity. The results are shown in Figure 1. Increasing the antibody concentration up to 20 ng mL^−1^ caused an increase in the SPRi signal, but above that level, there was no further rise in the SPRi signal. This is due to saturation of the active points on the biosensor surface. Therefore, a concentration of 20 ng mL^−1^ of antibody was selected as the optimal value for further experiments. The limit of detection (LOD) in this experiment was calculated by formula 3.3 SD/A (A- slope of the calibration curve) and it was 0.905 ng/mL.

### 3.2. Dependence of the Biosensor on FSH Activity—Calibration Graph

**Figure 2:**
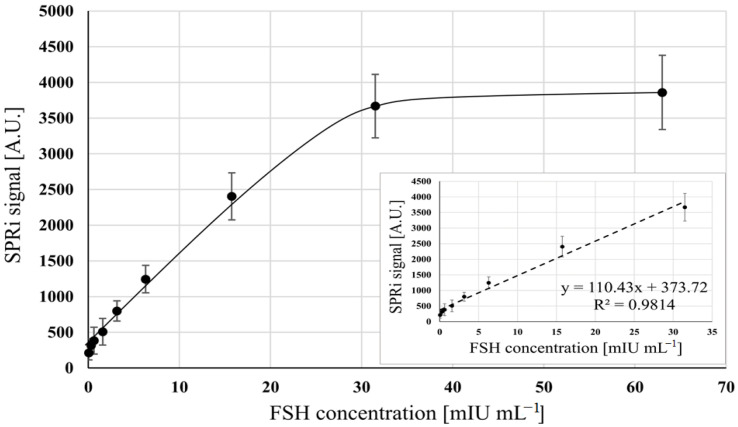
Dependence of the analytical response of the biosensor on the FSH activity. Applied mouse monoclonal anti-FSH antibody concentration: 20 ng mL^−1^, pH 7.4. Error bars were calculated for 12 independent measurements for each concentration at a 95% confidence level.

Under optimized conditions (20 ng mL^−1^ of the antibody), the dependence of the analytical signal on FSH activity was investigated. The investigated range of FSH activities was between 0.05 and 31.5 mIU mL^−1^. The experiments were performed in a PBS buffer (pH 7.4). The results are shown in Figure 2. The resulting graph is Γ-shaped, with the formation of a plateau, corresponding to saturation of the active points of the biosensor. A linear dependence was observed up to an activity of approximately 20 mIU mL^−1^, with R2 0.9987. The linear response range, therefore, lies between 0.05 and 20 mIU mL^−1^.

### 3.3. Precision and Recovery

The precision and recovery of the biosensor were checked in a series of experiments in a PBS buffer with different spikes of FSH between 0.063 mIU mL^−1^ and 31.5 mIU mL^−1^. The results are shown in Table 1. The lowest spike is above LOD (0.024 mIU mL^−1^) and below LOQ (0.080 mIU mL^−1^), while the highest spike is on the edge of linearity.

Generally, the results of Table 2 show that the precision is better for higher FSH spikes and is quite satisfactory. Recoveries are between 94% and 108% and are acceptable.

### 3.4. Selectivity

The selectivity of the biosensor is a decisive factor for its application in the determination of FSH in real samples. Human albumin and three hormones—luteinising hormone (LH), thyroid stimulating hormone (TSH) and testosterone—were selected as potential interferents. Hormones LH, TSH and FSH are composed of the same α subunit; therefore, they may exhibit similar biological activity. Testosterone, as a sex hormone that regulates FSH secretion, may also influence the selectivity of the method [30]. The concentration of albumin in the blood is at a very high level; therefore, it has a possible impact on FSH determination [31]. Tests were performed in a PBS buffer (pH 7.4) with the ratio of FSH to interferent equal to 1:1 and 1:10. Albumin, which is a protein present in blood at a high concentration, was additionally investigated at a ratio of 1:100. The results are shown in Table 3.

The results show that none of the investigated potential interferents have a significant influence on the determination of FSH with the developed biosensor. Recoveries are in a range between 97.5% and 105%, slightly better than the recoveries shown in Table 1. RSD does not exceed 10%. Thus, the selectivity of the biosensor is satisfactory.

### 3.5. Recovery of the FSH Spike in Plasma Sample

The final stage of investigation of the biosensor’s potential consisted of experiments performed with real samples. Real samples contain all potential interferents. Therefore, determination of an FSH spike should reveal the influence of these interferents. However, the FSH activity in non-spiked samples should also be taken into consideration. FSH activity was determined in three randomly selected human plasma samples. These samples, used without dilution, were spiked with 3.15 mIU mL^−1^ FSH and the recoveries were determined. The results are shown in Table 4.

The results show that the recoveries are satisfactory, and the precision of FSH determination is satisfactory and better than in the model investigation (see Table 1).

### 3.6. Validation of the Biosensor by Comparison with a Reference Method

**Figure 3:**
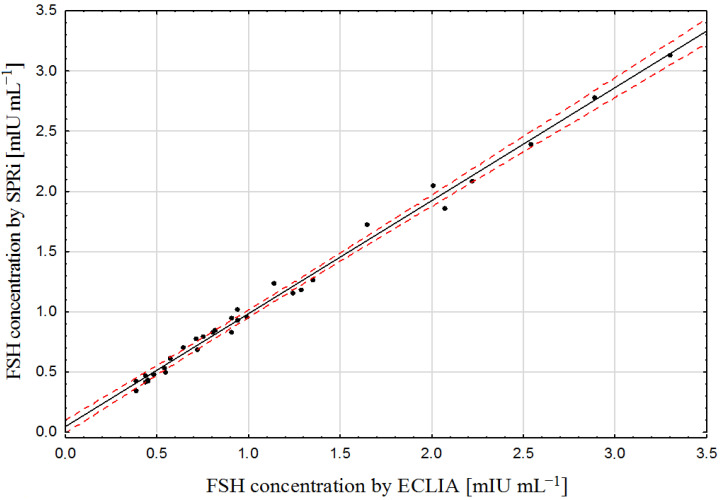
Pearson correlation of FSH determination in plasma samples (*n* = 22) using ECLIA and the developed biosensor. The plot of correlation (**―**), confidence interval (- - -).

Validation of the biosensor was performed by the simultaneous determination of FSH in 22 samples of plasma using the electrochemiluminescence assay (ECLIA) and the developed biosensor. The measurements with ECLIA were made in a routine laboratory. The results are shown in Figure 3.

The R^2^ value of 0.940 confirms the high level of similarity of the results of the two compared methods.

## 4. Discussion

The determination of serous FSH concentration is significant as an alternative to testicular biopsy in boys suffering from cryptorchidism after orchidopexy, and for the determination of the menopausal stage in woman. Unfortunately, there is no agreement as to what level is characteristic for the menopausal period, with very divergent levels being reported from 50–65 mIU mL^−1^ [15] to 70–124 mIU mL^−1^ [18]. Additionally, Itoh et al. reported that the results may depend on the analytical method used [14]. All methods for FSH determination used to date involve the use of labels: radioactive, luminescent or electroluminescent. The method proposed in this paper is a label-free alternative to those methods. Additionally, the array SPRi results for FSH in a series of plasma samples show good agreement with the results obtained by the electrochemiluminescence method (see Figure 3), which is strong evidence that the results of both methods are correct. Additionally, our measured serum FSH levels in boys with cryptorchidism (0.35–3.31 mIU mL^−1^) are similar to those obtained by Christiansen et al. (0.25–3.73 mIU mL^−1^) [30].

The range of linear response of the developed method makes it well suited to FSH determination in serum or plasma. Linearity was obtained between 0.05 and 20 mIU mL^−1^. However, the true accessible range is from 0.08 mIU mL^−1^ (LOQ) to 20 mIU mL^−1^. Most serum or plasma samples can be applied without dilution, but samples from menopausal women should be diluted with a PBS buffer. The precision of measurement is between 3.2% and 13.1% for model samples, depending on the FSH spike, and between 3.7% and 5.6% for spiked plasma samples. These are quite good levels of precision. Recoveries range from 94% to 108% for model FSH samples and from 102% to 103% for plasma spiked with FSH. These recoveries can be regarded as acceptable. In our study, a lower detection limit (0.024 mIU mL^−1^) was achieved compared to those of the methods presented in Table 1 and the standard method used for validation (ECLIA) (0.100 mIU mL^−1^).

The selectivity of the biosensor is a decisive factor for its application in the determination of FSH in real samples. Three hormones related to FSH were investigated as potential interferents: luteinizing hormone (LU), thyroid stimulating hormone (TSH) and testosterone. FSH, LU and TSH belong to the family of glycoprotein hormones, and are homologues in terms of sequence, structure, and function [32]. Both FSH and LU are involved in the regulation of testosterone level [18,30]. Additionally, albumin—the protein occurring in serum at the highest concentrations [31]—was also investigated as a potential interferent. The lack of significant influence of these hormones, when present in excess, on the measurement results shows the good selectivity of the developed biosensor. However, the best test of the sensor’s selectivity is FSH determination in plasma samples, where all potential interferents are present. Spikes of FSH in plasma samples are recovered well, and the precision is the same as or better than in the model investigations.

The SPR immunoassay method in the classic version was used to detect FSH [21]. SPR measurements in a flow system were performed. A commercial SPR biosensor was employed for SPR measurements. A binding inhibition immunoassay format was used for the detection of FSH. The hormone was covalently attached to the sensor surface using amino coupling between a carboxyl group end in the SAM (self-assembled monolayer) and amino groups in the protein. For this FSH assay, the detection limit obtained had a higher value (LOD = 29 mIU mL^−1^) than that of the developed SPRI biosensor. The measurement was performed using the two flow cells. The sample injection volume was 220 µL.

A significant advantage of the developed biosensor is the small sample of blood plasma required for a single measurement. Only 3 µL of plasma is applied to the biosensor. Such small sample volume enables the performance of many different determinations from a single blood sample. Although the experiments with real samples used blood plasma, it is likely that very similar results would be obtained with blood serum. An additional advantage of this biosensor is the possibility of making simultaneous determinations from multiple samples (usually nine). These two factors reduce the cost and time of a single measurement of FSH in blood plasma. Array SPRi biosensors have been successfully used to determine biomolecules not only in blood, but also in urine [23,33] and saliva [34,35]. However, the potential application of the developed biosensor in the medical field beyond the determination of FSH concentration in blood plasma requires further research. Such studies may provide information on the cost effectiveness and practicality of using this biosensor in clinical settings.

This biosensor is therefore a promising new tool for liquid biopsy using the array SPRi technique.

## Figures and Tables

**Table 1:** The analytical parameters of exemplary methods for determining FSH.

Methods	LOD	Sensitivity	References
immunoradiometric assay	n/d	1.92 mIU mL^−1^	[14]
double antibody radioimmunoassay	n/d	0.42 mIU mL^−1^	[14]
fluoroimmunoassay	0.05 mIU mL^−1^	n/d	[15]
electrochemiluminescence immunoassay	0.05 mIU mL^−1^	n/d	[4]
capillary electrophoresis with enhanced chemiluminescence detection	0.06 mIU mL^−1^	n/d	[19]
stochastic biosensor	0.014 mIU mL^−1^	n/d	[20]
SPR immunoassay	29 mIU mL^−1^	28 pM	[21]
SPRi biosensor	0.024 mIU mL^−1^	n/d	This work

n/d—no data.

**Table 2:** Precision and recovery of the biosensor.

FSH Spike (mIU mL^−1^)	Found (mIU mL^−1^)	SD (mIU mL^−1^)	CV (%)	Recovery (%)
0.063	0.059	0.008	13.1	94
0.315	0.337	0.029	8.6	107
1.58	1.71	0.183	10.7	108
3.15	3.10	0.265	8.6	98
6.30	6.13	0.225	3.7	97
31.5	32.0	1.02	3.2	103

**Table 3:** Selectivity of the biosensor.

FSH Spike (mIU mL^−1^)	Ratio FSH vs. Interferent	Found (mIU mL^−1^)	SD (mIU mL^−1^)	CV (%)	Recovery (%)
Albumin					
15.8	1:1	15.5	1.34	8.64	98.3
	1:10	16.8	1.32	7.86	107
	1:100	15.9	0.92	5.79	101
LH					
15.8	1:1	16.6	0.37	2.23	105
	1:10	15.7	0.52	3.31	99.8
TSH					
15.8	1:1	16.0	0.55	3.43	101
	1:10	16.3	1.07	6.56	104
Testosterone					
15.8	1:1	15.3	1.44	9.41	97.5
	1:10	15.4	1.14	7.40	99.3

**Table 4:** Recovery of a spike of 3.15 mIU mL^−1^ FSH in a plasma sample as determined using the developed biosensor.

FSH in Plasma Sample (mIU mL^−1^)	Expected FSH Activity (mIU mL^−1^)	Found FSH Activity (mIU mL^−1^)	Recovery (%)	CV (%)
2.12 ± 0.214	5.27	5.43	103	5.6
0.573 ± 0.063	3.72	3.81	102	3.7
3.00 ± 0.113	6.16	6.26	102	4.1

## Data Availability

Data are contained within the article.

## Supplementary Materials

The following supporting information can be downloaded at: https://www.mdpi.com/article/10.3390/s23249686/s1, Figure S1: Scanning electron microscope images of the biosensor showing the process of layer formation on its surface.
