# Supplementary material for: An Array SPRi Biosensor for the Determination of Follicle-Stimulating Hormone in Blood Plasma
Source: Sensors (Basel). 2023 Dec 7;23(24):9686. doi: 10.3390/s23249686 (PMC10747586; doi:10.3390/s23249686)
Supplement: Supplementary file 1 [file sensors-23-09686-s001.zip › sensors-2731438-supplementary.pdf]

sensors

Supplementary Materials

An Array SPRi Biosensor for the Determination of Follicle-Stimulating Hormone in Blood Plasma

The control of formation of successive immunosensor layers.

During preparation of the biosensor, the surface was controlled by scanning electron microscope (SEM). The SEM measurements were performed using an INSPEC S60 microscope from FEI (Hillsboro, OR, USA). The microscope was equipped with a tungsten electron source and a backscattered electron detector were. A voltage of 12.5 or 15 kV and magnification of 100,000× were used for the tests.

The results are shown in Figure S1.

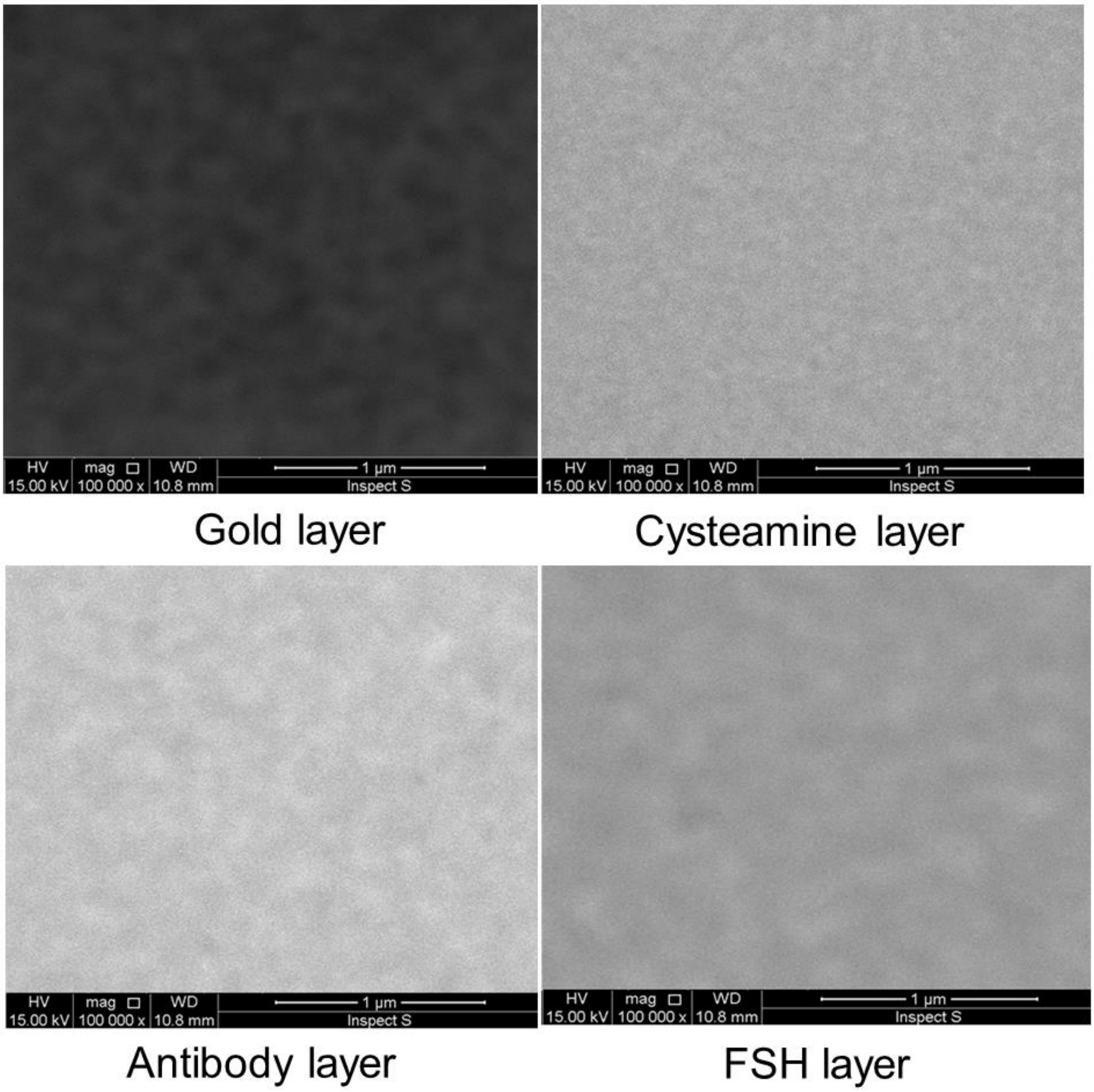

Figure S1. Scanning electron microscope images of the biosensor showing the process of layer formation on its surface.
